# Evaluation of Bond Strength of Four Different Root Canal Sealers

**DOI:** 10.3390/ma15144966

**Published:** 2022-07-17

**Authors:** Sanda Ileana Cimpean, Adela Loredana Colceriu Burtea, Radu Stefan Chiorean, Mircea Cristian Dudescu, Aurora Antoniac, Alina Robu, Radu Septimiu Campian, Lucia Iacobina Timis

**Affiliations:** 1 Faculty of Dentistry, Iuliu Hatieganu University of Medicine and Pharmacy, 8 Victor Babes Str., 400000 Cluj Napoca, Romania; sandacimpean@gmail.com (S.I.C.); rcampian@email.com (R.S.C.); t.lucia@yahoo.com (L.I.T.); 2 Faculty of Automotive, Mechatronics and Mechanical Engineering, Technical University of Cluj-Napoca, 103-105 Bdul. Muncii, 400641 Cluj-Napoca, Romania; radu.chiorean@rezi.utcluj.ro (R.S.C.); mircea.dudescu@rezi.utcluj.ro (M.C.D.); 3 Faculty of Material Science and Engineering, University Politehnica of Bucharest, 313 Splaiul Independentei, District 6, 060042 Bucharest, Romania; antoniac.aurora@gmail.com (A.A.); alinarobu2021@gmail.com (A.R.)

**Keywords:** push-out, endodontic sealer, SEM

## Abstract

The purposes of the study were to evaluate the influence of the sealer’s chemical composition on the interfacial strength between root canal dentin and root filling material, for two different classes of endodontic sealers, and to assess their failure modes. *Methods*: Forty extracted single-rooted teeth were randomly divided into four groups using the following endodontic sealers: RealSeal SE and Resilon (RSSE); EndoSequence BC sealer and BC Point (EBCS); Endoseal MTA and gutta-percha (EDS); Bioroot RCS and gutta-percha (BRS). Teeth were embedded in acrylic resin, and the roots were sectioned horizontally into 1 mm slices. For each slice, the perimeter was measured. A push-out test was performed using an Instron universal testing machine. For each sample, bond strength was calculated. Specimens were examined by SEM investigation in order to analyze the dentin–sealer–core interface. Results were assessed using analysis of variance (ANOVA) and Tukey and Bonferroni test. *Results*: Statistical analysis revealed that EDS and gutta-percha had significantly higher resistance to dislodgement compared to the other three groups (*p* < 0.05). EBCS and BC Point showed significantly greater push-out bond strength values compared to RSSE and Resilon (*p* < 0.05). *Conclusions*: Bioceramic endodontic sealers showed a higher bond strength to root dentin than methacrylate resin-based endodontic sealer.

## 1. Introduction

The goal of root canal obturation is to obtain an adequate seal between the dentinal walls and the endodontic filling materials. The endodontic obturation must entomb residual bacteria and behave as a barrier that would prevent infection or reinfection of the root canal system [1]. One of the main causes of endodontic treatment failure is bacterial leakage. Fluid passage between the root canal and either oral cavity or periapical tissue can affect the periapical health.

Root canal filling is obtained by combining a solid filling material such as gutta-percha or Resilon, which functions as a core, with a fluid endodontic sealer. The role of the sealer is to fix or cement the core obturation material into the root canal, filling the voids and the lateral or accessory canals. Ideally, the sealer should adhere both to dentin and to solid filling material, and it should be dimensionally stable, thus ensuring a hermetic seal of the root canal [2]. It has been observed that a 1% shrinkage of the root canal sealer can produce a gap at the sealer–gutta percha and/or sealer–dentin interface, large enough for bacterial penetration [3].

Root canal instrumentation decreases tooth resistance; therefore, endodontic obturation should create a mechanically homogeneous unit with root dentin, thus accomplishing an endodontic monoblock [4].

The chemical composition of endodontic sealers can influence their adhesion to root dentine and, hence, the effectiveness of root canal obturation. To reduce bacterial leakage after endodontic therapy, efforts were made to improve the sealer’s adhesion to root canal dentin. Consequently, materials with adhesive properties were developed.

Real Seal SE (SybronEndo, Orange, CA, USA) is a dual-cure self-etch methacrylate resin-based endodontic sealer that bonds covalently to the dentin surface. It is used in conjunction with Resilon cones, which consist of a basis of polycaprolactone and a dimethacrylate resin with radiopaque fillers. The cone was designed to bond chemically to resin-based sealers, thus forming a secondary monoblock [5]. Regarding the ability of Real Seal SE Resilon to create a monoblock, the results of various studies are contradictory; several studies showed promising results, [6] while others showed the opposite [7].

Due to the excellent sealing ability of calcium silicate-based cements, such as mineral trioxide aggregate, several calcium silicate-based sealers were introduced in endodontics in the last years [8]. These materials seal the root canal by chemical bonding, forming a layer of hydroxyapatite on the dentin surface, and by micromechanical interaction through tag-like structures [9,10]. In addition, these sealers possess high alkalinity, increased biocompatibility, low shrinkage, and a good mineralization activity [11,12]. Bioroot RCS (Septodont, St. Maur-des-Fossés, France) is a two-component bioceramic endodontic sealer with internal water supply. When in contact with dentinal fluid, due to its hydrophilic properties, a calcium phosphate phase is formed, inducing mineral plugs within the dentinal tubules [13,14]. It has also been proven to stimulate the metabolism of human periodontal ligament cells [15,16].

EndoSequence BC sealer (Brasseler USA, Savannah, GA, USA) is a premixed, hydrophilic, hydraulic bioceramic sealer, with good dimensional stability and no shrinkage after setting [17]. It is a bioactive material with satisfactory biological and antimicrobial properties due to its content of calcium silicates, calcium phosphate, calcium hydroxide, and zirconium oxide. According to the manufacturer’s instructions, this sealer is designed to be used in combination with gutta-percha points that are impregnated and coated with bioceramic particles (BC Point) [18].

Endoseal MTA (Maruchi, Wonju, Korea) is a pozzolan-based premixed bioceramic endodontic sealer, with excellent biological and cementation properties [19]. Previous studies reported that Endoseal MTA is able to produce an intratubular biomineralization for a depth of up to 350–400 μm in dentinal tubules [20], demonstrating good sealing ability [21].

Regarding the sealing ability of bioceramic sealers, previous studies revealed significantly deeper dentinal tubule penetration, less dye leakage, and higher push-out bond strength when compared to other sealers [22,23,24]. Neelankantan et al. demonstrated a close inverse correlation between leakage and root filling bond strength; therefore, the measurement of filling bond strength (FBS) allows the appreciation of the sealing ability [25].

Resistance to dislocation for root canal filling materials has been evaluated in vitro using the push-out test [26]. This test has the advantage of developing a uniform shear strength while the material is tested within the root canal [27]. Dislocation of the filling material can either occur when its bond to dentin breaks, causing an adhesive failure, or when the filling material suffers internal fractures, resulting in a cohesive failure.

Choosing the best endodontic sealer for a specific clinical case is strongly influenced by the comprehension of their composition, their properties, and indications [2]. Both methacrylate-based sealers and bioceramic sealers claim good adhesion to dentinal surface, although appropriate testing methods are necessary for a proper comparison.

The primary purpose of the present study was to evaluate the influence of the sealer’s chemical composition on the interfacial strength between root canal dentin and root filling material, for two different classes of endodontic sealers (methacrylate resin sealer and bioceramic sealers). The secondary objective of this study was to assess the failure modes of these materials. The null hypothesis tested was that there is no difference in bond strength among the different endodontic sealers tested.

## 2. Materials and Methods

### 2.1. Tooth Selection

Forty single-rooted human teeth, extracted for periodontal reasons, were selected. Approval for this study was obtained from the local committee of ethics (no 16/03.02.2022), and all subjects gave their informed consent for using the extracted teeth. Prior to their use, teeth were evaluated under an endodontic microscope (Alltion AM-6000, Wuzhou, China) and by radiological examination. The endodontic microscope was used to examine the exterior surface of the extracted teeth, as well as the location of the apical foramen three dimensionally. Investigation was performed in order to visualize the possible carious lesions, cracks, fractures, or resorption defects of the root. To observe these small details, a magnification of 14× was used. The standardized radiographs were taken from the buccal and proximal aspects in order to determine the presence of single canal, mature apical foramen, root canal curvature, previous root canal treatment, or internal resorption, using X-Mind^TM^ DC (Satelec, Merignac, France). For the radiologic examination, the teeth were placed in horizontal position with the long axis parallel to the sensor and the central X-ray beam perpendicular to the long axis of the root canal. The root curvature was quantified using Schneider criteria [28] The assessment of the circular shape of the root canal was performed radiologically, by determining the ratio between the buccal–oral and the mesio-distal diameter, in the coronal and middle third of the canal where it was more visible radiologically. In the apical third, the root canals normally had a round shape. Only the teeth with a mesio-distal diameter close to the buccal–oral one were included. Inclusion and exclusion criteria are presented in Table 1.

### 2.2. Sample Preparation

After extraction, each tooth was immersed for 3 h in a 5.25% sodium hypochlorite solution (Cerkamed Company, Stalowa Wola, Poland), for a proper disinfection. Tissue debris and calcified deposits were removed with ultrasonic scalers (Newtron Booster, Satelec-Acteon, Merignac, France). Teeth were stored in saline solution until their preparation.

Access cavities were prepared in a standard manner using round diamond burs (Jota, Switzerland) and Endo Access Bur (Dentsply Sirona, Ballaigues, Switzerland). The working length was determined visually, using a #10 K-file (Dentsply-Maillefer, Ballaigues, Switzerland) until it was visible at the apical foramen and subtracting 0.5 mm from the total measured length. All teeth were instrumented at the working length using a Wave One reciprocating system (Dentsply-Maillefer, Ballaigues, Switzerland), choosing Wave One Large as the master apical file (40/08). Root canals were irrigated after the use of each endodontic file with 1 mL of 5.25% sodium hypochlorite (NaOCl) (Cloraxid, Cerkamed, Stalowa Wola, Poland), using a 5 mL syringe and 30-gauge side-vented needle (Cerkamed, Stalowa Wola, Poland). Final irrigation was performed with 3 mL of 5.25% NaOCl, followed by 1 mL of 17% EDTA (MD Cleanser, Meta Biomed, Cheongwon-gun, Korea) as the final irrigant and dried with paper points. All preparation procedures were carried out by the same operator.

Subsequently, the teeth were distributed randomly into four groups and obturated using two different obturation techniques, according to the producers’ indication, in order to replicate better the clinical situation as follows:

Group 1: RealSeal SE (SybronEndo, Orange, CA, USA) and 0.06 taper Resilon cone (SybronEndo, Orange, CA, USA), using warm vertical condensation technique (RSSE);

Group 2: EndoSequence BC sealer (Brasseler USA, Savannah, GA, USA) and 0.06 taper BC Point (Brasseler USA, Savannah, GA, USA), using single-cone technique (EBCS);

Group 3: Endoseal MTA (Maruchi, Wonju, Korea) and Wave One Large gutta-percha cone (Dentsply-Maillefer, Ballaigues, Switzerland), using single-cone technique (EDS);

Group 4: Bioroot RCS (Septodont, St.Maur-des-Fossés, France) and Wave One Large gutta-percha cone (Dentsply-Maillefer, Ballaigues, Switzerland), using single-cone technique (BRS);

Chemical composition of tested endodontic sealers is presented in Table 2.

The master cone size #40 was checked prior to obturation, in order to feel the tug-back sensation. Following root obturation, coronal cavities were restored with light-curing composite resin (Herculite XRV (Kerr Corporation, Orange, CA, USA). The teeth were maintained at 370 °C for 7 days, in a humid environment, to allow the sealer to set. All obturation procedures were performed by the same operator, an endodontist with more than 25 years of experience. Accordingly, the same force was used for all teeth that were obturated with the single-cone technique.

**Figure 1:**
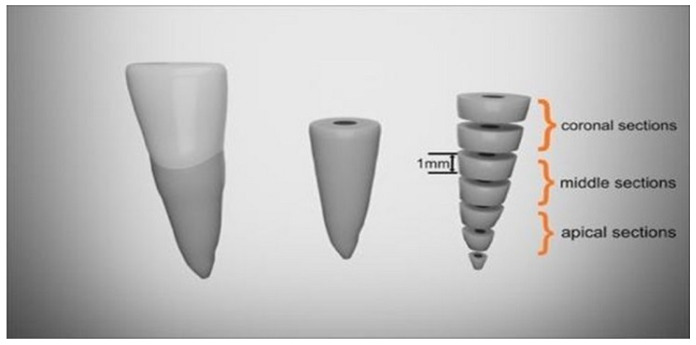
Schematic representation of horizontal cross-sections of the root.

After 7 days, teeth were embedded into acrylic resin (Duracryl plus, Spofa Dental, Jičín, Czech Republic) in a vertical position and then sectioned, perpendicular to their long axis, with a water-cooled diamond blade disc (Ø125 × 0.35 × 12.7 mm Isomet, Buehler, Lake Bluff, IL, USA), using an Isomet machine (Buehler, Lake Bluff, IL, USA) [19] Each root was sectioned horizontally into 1 mm thick slices starting at 1 mm from apex, up to the cementoenamel junction (Figure 1).

**Figure 2:**
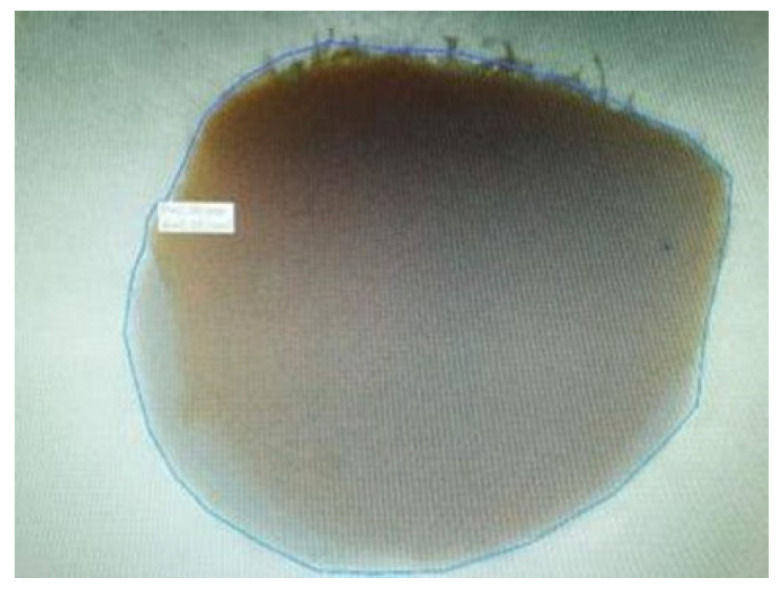
Perimeter measurement.

A marker was used to mark and later recognize the apical surface of each sample. A digital caliper was used to measure the actual thickness of each sample with a precision of 1 µm. Both sides of each sample were examined under Olympus CKX 41 microscope (Olympus, Tokyo, Japan), at 10xmagnification rate and for those with circular shape and without voids. The perimeter was measured using Quick Photo MICRO 3.0 software (Figure 2). Following sectioning of the roots, a total of six slices of 1 mm thickness were obtained for each tooth (10 teeth per group). This resulted in a total of 60 specimens per group. The sample size was calculated with a power calculation for one-way ANOVA (power = 0.8; effect size = 0.25; significance level = 0.05; number of groups = 4; SD = 4) resulting in the recommended sample size of a minimum 37 samples per group. When the sections showed an oval canal or canal with isthmuses, the sections of that tooth were removed and replaced by preparing and filling additional teeth. This occurred for two teeth.

### 2.3. Methods for Mechanical Testing

**Figure 3:**
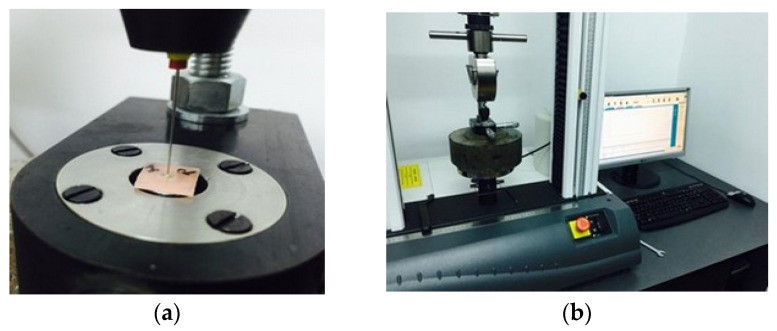
Push-out test performed in a coronal direction by the universal testing machine (**a**). The force required for dislodgement was recorded (**b**).

This assay was a modification of the technique used by several investigators [19,21,25,26,29]. Resistance to dislodgement was evaluated using stainless-steel pluggers with different diameters tips, ranging from 0.3 mm to 0.8 mm, closely matching the diameter of the obturation. The force applied acted in the apical–coronal direction, to avoid any constriction caused by root canal taper. The chosen plugger was positioned such that the root canal obturation was covered as much as possible, without touching the surrounding root canal walls. The push-out force was generated using a universal testing machine (Instron modell 3366, Instron Corp., Nowood, MA, USA), at a crosshead speed of 0.5 mm/min, until bond failure occurred [19,29]. For this test, the specimens were placed on a cylindrical tube with a biaxial micrometric screw positioning system, to facilitate the alignment with the punch and the free dislodgement of the root filling material. The maximum force applied to root canal obturation before debonding was recorded in Newtons (Figure 3).

Bond strength was defined as the force value per unit area (similarly to a mechanical shear stress; expressed in MPa) and was calculated according to the following formula [25,26]:

τ (MPa) = F/Af,(1)

where F represents the maximum force, and Af is the conventional adhesion area. Af was considered a cylindrical surface area, due to the small thickness of the sample and the small difference in taper between the coronal and apical side.

Af (mm^2^) = (Pa + Pc)/2 × g, (2)

where Pa represents the apical perimeter, Pc is the coronal perimeter, and g is the height of the slice.

### 2.4. Microscopical Methods for Interface and Failure Evaluation

**Figure 4:**
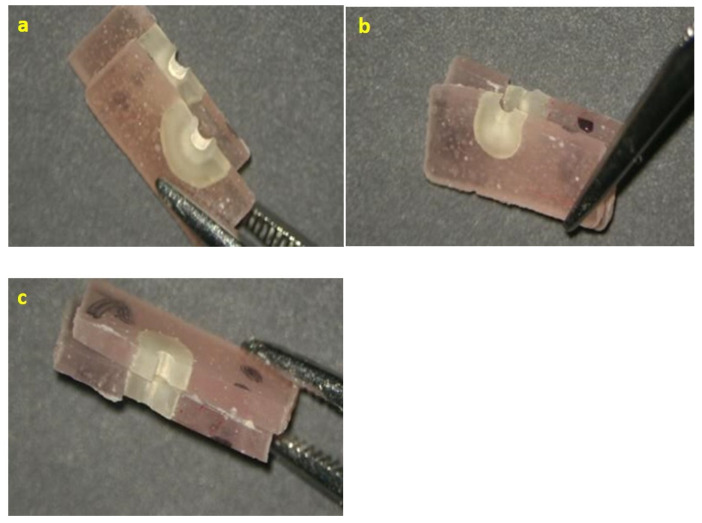
Representative failure modes for tested endodontic sealers: (**a**) cohesive EBCS; (**b**) adhesive RSSE; (**c**) mixed BRS.

In order to determine the failure pattern, the sections were cut longitudinally, using a diamond disc, and the interface root canal dentin/obturation material was examined by two blinded evaluators, under magnification, using an endodontic microscope (Alltion AM-6000, Wuzhou, China). This microscope allowed the examination of both the horizontal and the vertical surfaces (Figure 4).

The evaluation was conducted using the classification of Stelzer R [29] according to which we considered the failure to be adhesive, when less than 25% of the dentin surface was covered by sealer, cohesive, when more than 75% of the dentin surface was covered by sealer, or mixed, when the dentin surface covered by sealer was between 25% and 75%.

### 2.5. Scanning Electron Microscopy (SEM) Evaluation

Three random specimens from each group were selected for SEM investigation (*n* = 12) and examined by a QUANTA INSPECT F Scanning Electron Microscope (FEI Company, Eindhoven, the Netherlands), working at an acceleration voltage of up to 30 kV and equipped with an energy-dispersive X-ray spectrometer detector (EDAX) with a 132 eV resolution at Mn-Kα. Specimens were dried, mounted on aluminum stubs, placed in sealed glass vials with silica, and sputter-coated with gold in a vacuum chamber. In order to analyze the dentin–sealer–core interface, serial SEM photomicrographs were taken at different magnifications (40× and 4000×). Two evaluations were performed for each photomicrograph.

### 2.6. Statistical Analysis

The normality of statistical distributions was assessed visually using rainclouds (combining a cloud of points with a boxplot and a one-sided violin plot) and Q–Q plots.

One-way analysis of variance (ANOVA) for independent samples was used to test for differences among groups defined by endodontic filling materials. The homogeneity of variance was assessed by using Levene’s test. Pairwise comparisons between groups were assessed using bootstrapped post hoc standard test with Tukey and Bonferroni corrections. The level of statistical significance was set to α = 0.05. All graphics and analyses were performed using JASP (JASP Team 2021, JASP Version 0.16).

## 3. Results

### 3.1. Results of Mechanical Testing

The mean push-out test values and standard deviations are presented in Table 3.

**Figure 5:**
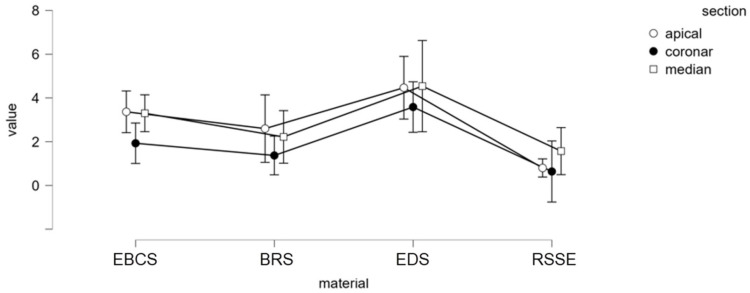
Mean push-out bond strength (MPa) for tested endodontic sealers.

Significant differences were observed among the endodontic materials (Figure 5).

Pairwise comparisons between groups (Tukey’s test and Bonferroni corrections) revealed that group 3 (EDS and gutta-percha) had significantly higher dislodgement resistance than the other three groups (*p* < 0.05). EBCS and BC Point showed significantly greater push-out bond strength values compared to RSSE and Resilon (*p* < 0.05). There were no significant differences between EBCS and BRS nor between RSSE and BRS.

When comparing the bond strength values for each third of the root, middle and apical samples had significantly higher values compared to coronal samples (*p* < 0.05). There were no significant differences between the push-out bond strengths in apical and middle specimens. (Table 4).

**Figure 6:**
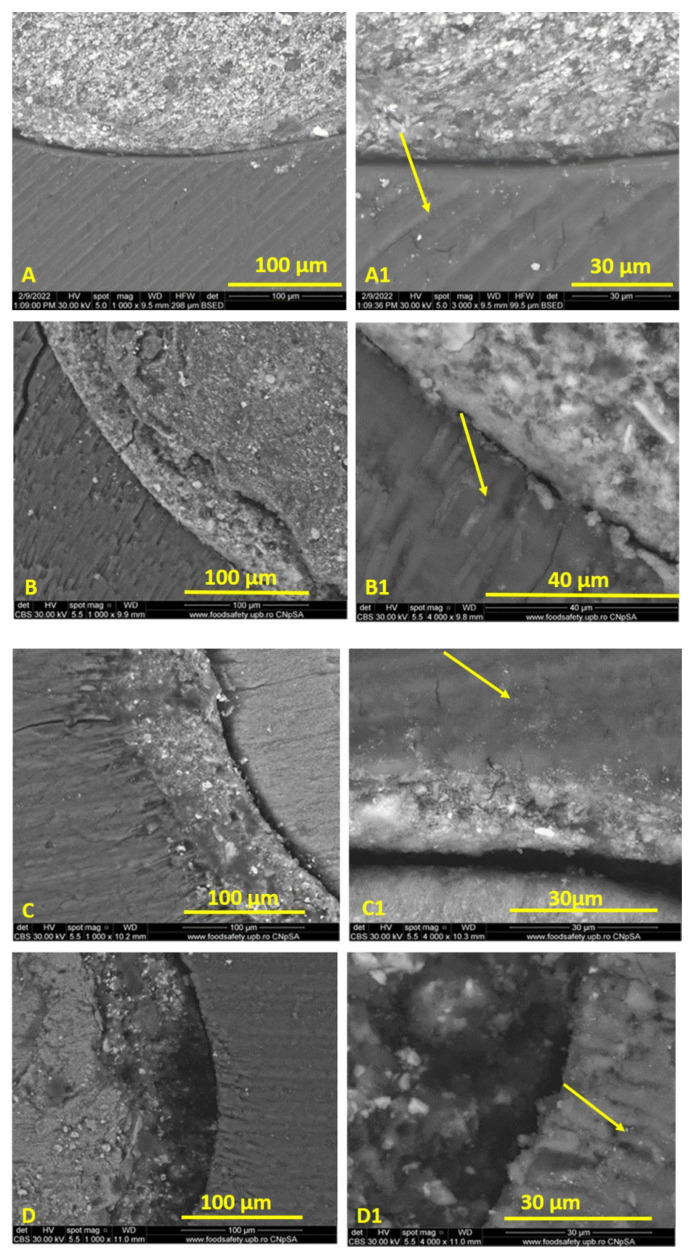
Representative images of interfacial failure and dentine tubule penetration for tested endodontic sealers. RSSE—adhesive failure (**A**,**A1**); EBCS—cohesive failure (**B**,**B1**); EDS—cohesive failure (**C**,**C1**); BRS—adhesive failure (**D**,**D1**).

The results of mode of failure analysis are presented in Table 5 and Figure 4. For both EDS and EBCS, there was a predominance of cohesive failure modes, whereas the sealers RSSE and BRS principally displayed adhesive failure modes (Figure 6A–C).

### 3.2. Results of Scanning Electron Microscopy (SEM) Evaluation

The scanning electron micrographs revealed that BRS had an area of structureless morphology at the sealer–dentine interface, characterized by the absence of large particles and by the inclusion of small particles (Figure 6D). EDS and EBCS exhibited the same microstructure, both inside the sealer mass and at the contact with the dentin of the canal walls (Figure 6B,C). SEM images of the dentin–sealer interface revealed a modified-looking dentin band for all bioceramic sealers, due to the presence of tag-like structures within dentinal tubules (Figure 6B1–D1).

## 4. Discussion

Many studies have suggested that a chemical bond to root dentine would provide a three-dimensional seal of the root canal, improving the resistance to dislocation of the endodontic obturation(s). The primary purpose of the present study was to evaluate the manner in which the chemical composition of two different classes of endodontic sealers influenced the push-out bond strength. Both classes of materials claim to achieve a chemical adhesion to the dentinal surface. Significant differences in bond strength were observed between tested root canal sealers; therefore, the null hypothesis was rejected.

The push-out bond strength test is widely used when investigating the sealing ability of endodontic obturation materials. It is considered a valuable method both for evaluating the adhesion strength of endodontic sealers to dentinal walls or to core materials and for classifying the materials according to their bonding capacity [30,31]. The accuracy of this test may be influenced by several variables such as root canal preparation, root canal obturation, plugger diameter, sample orientation, specimen thicknesses, and core material stiffness. In the present study, the use of a #40 file with 8% conicity allowed uniform and reproductible instrumentation of the canal walls, ensuring equal shape and size of the prepared root canals.

The obturation techniques were chosen according to the manufacturers’ indications and the techniques used in clinical practice, in order to obtain optimal results for both classes of materials. Although warm vertical compaction is considered superior to the single-cone technique, it is not indicated for bioceramic sealers tested in this study, as the heat would change these materials’ adhesion properties, thereby affecting the results [18].

The most important parameter appears to be the plugger diameter–specimen diameter ratio. Studies conducted by Pane et al. and Nagas et al. demonstrated that a ratio between 70% to 90% would not affect the bond strength, while a ratio of less than 55% would present lower values as an outcome [30,32]. Chen [33] stated that the diameter of the plugger should be 0.85 times smaller than the diameter of the root canal obturation, but large enough to prevent its notching into the material’s surface. In our study, pluggers of different diameters (0.3–0.8 mm) were used to closely match the diameter of the root filling material, for each tested sample.

According to Chen et al. [33], the specimen thickness should be 0.6 times larger than the diameter of the obturation, in order to prevent it from influencing the push-out bond strength value. In our study, specimen thickness was 1 mm and the diameter of the root canal obturation ranged between 0.4 mm and 1.14 mm.

The present study revealed that the push-out bond strengths of EDS and gutta-percha were significantly superior to the other sealers. All three calcium silicate-based sealers (EBCS, EDS, and BRS) scored significantly higher than RSSE, probably due to intrafibrillar apatite deposition [21,34,35]. The differences in chemical composition of the bioceramic sealers may influence their interaction with the root dentin, having a significant impact on their adhesion [19,36].

Apatite formation is directly proportional to the quantity of calcium ions available; therefore, an increased release of calcium ions may result in an increased bioactivity and mineral deposition at sealer/dentin interface [13]. This may explain the differences between the values regarding the resistance to dislodgement, for the three tested calcium silicate endodontic sealers.

In our study, the displacement of the obturation at the sealer–core interface occurred for all tested sealers in different proportions. De-Deus et al. [37] demonstrated that EDS displayed increased adhesion to gutta-percha compared to EBCS, possibly due to its distinctive composition: a pozzolan-based sealer that reacts chemically with calcium hydroxide in the presence of water, resulting in compounds with adhesive properties. This may explain the superior values of push-out bond strengths obtained when using EDS.

Nevertheless, our study also revealed good values during the push-out test for EBCS; the results agree with studies performed by Pawar et al. [38] and Gade et al. [39]. Likewise, Donnermeyer’s study [34] revealed that monophasic calcium silicate-based sealers displayed better values for push-out tests than two-component sealers, and BRS was proven to possess inferior POBS (2.31 MPa versus 3.52 MPa) compared to Total Fill BC Sealer, which corroborates the results of our study. The same results were reported by Falakaloğlu et al. [40].

However, conflicting results were reported by Retana-Lobo et al. [35] who outlined better results for BRS (3.522 MPa) compared to EBCS (3.223 MPa) when root canal obturation was performed without GP, but with no significant statistical difference when the canal was obturated with sealer and GP cone.

Lin [21] presented comparative data regarding the dislodgement resistance of EDS and BRS. The sealers showed similar bond strength values before and after artificial aging, in contrast to our study results.

As stated by the manufacturer, RSSE can provide a good adhesion to radicular dentin, due to the resin tags that penetrate dentinal tubules. At the same time, the sealer creates a chemical bond to the Resilon cone, due to their homogeneous chemical composition, generating a monoblock system inside the root canal. In our study, RSSE presented the weakest adhesion force to the dentinal surface (1.059 ± 1.240). These values are in concordance with results reported by other studies [29,41,42]. Specifically, in the study released by Stelzer et al. [29], bond strength values ranged between 0.91 ± 0.64 and 1.28 ± 0.60 when hypochlorite, EDTA, or chlorhexidine were used for the final irrigation, with higher levels for hypochlorite followed by saline solution (2.35 ± 0.49). In our study, the final irrigation was carried out using NaOCl, followed by EDTA, to eliminate the smear layer and open the dentinal tubules, which, according to Stelzer, would reduce the dentinal surface required for sealer retention. The same effect was observed in Miletic’s study [41], where removal of the smear layer using PIPS diminished bond strength values for RSSE. In the absence of the smear layer, RSSE’s pH of 3.9 is not sufficiently strong to adequately demineralise the dentin, consequently affecting the hybrid layer development [43]. Ehsani [44] analyzed the effect of an erbium, chromium: yttrium–scandium–gallium–garnet laser on the push-out bond strength of RealSeal SE, indicating an increase in bond strength values for RSSE when the final irrigation was laser-activated.

Another explanation for obtaining these values for RSSE may be the low degree of conversion (DC) of the resin sealer, with a negative influence on its adhesive capacity and sealing ability [45]. The degree of conversion (for methacrylate-based resins) is affected by a series of factors, such as moisture degree or the temperature generated during curing [46].

Fuzinatto et al. [47] also achieved an increase in bond strength values after irrigating the root canal with ethanol. This was a consequence of ethanol’s ability to remove excess moisture from the dentin, thus preventing resin sub-polymerization and enhancing the bond between RSSE and root dentine. In our study, paper cones were used for drying the canal after the final irrigation with EDTA without the use of ethanol. This aspect can favor the maintenance of a certain degree of humidity at the level of the dentinal tubes which may have led to a decrease in dentine adhesion.

An increase in the polymerization rate significantly augments the contraction stress level [48]. This may be caused by the elevated temperature during warm vertical condensation technique when the pluger’s temperature reaches 150 °C. A high polymerization rate, alongside an extremely high C-factor in the root canal, may cause significant polymerization shrinkage stresses at the sealer–dentin interface, resulting in sealer debonding and leading to low values of push-out bond strength.

The push-out bond strengths in the apical and middle sections were significantly higher than those of the coronal sections for bioceramic sealers. There were no significant differences between the push-out bond strengths in the middle and apical specimens of bioceramic sealers. This may be due to a more accurate adaptation of the gutta-percha cone to the shape and size of the root canal in the median and apical thirds, leading to a better penetration of the sealer into the dentinal tubules.

The secondary aim of this study was to assess the failure modes of tested endodontic sealers. Regarding the mode of failures, EDS and EBCS predominantly reported cohesive failure modes (72.22% and 66.66%), consistent with previously reported findings [34,35]. BRS exhibited adhesive (63.15%) and cohesive (26.31%) failure modes. RSSE displayed adhesive and mixed failure modes, suggesting that the sealer adheres well to the Resilon core but less predictably to dentin. The mentioned findings are consistent with the results reported in previous studies [41,49]. The adhesive failures suggest an improper chemical bond between the sealer and root canal dentine. Furthermore, Nagas et al. [32] reported a positive correlation between resistance to dislocation and fractures inside the sealer; this aspect was also observed in our study.

Scanning electron microscopy (SEM) has been deployed in previous studies to highlight the sealer characterization, to analyze the sealer–dentin interface and sealer’s penetration into dentinal tubules [50,51,52,53,54,55]. In similar studies, a comparable number of samples were selected for SEM investigation [35]. In our study, SEM examination indicated that both EDS and EBCS presented the same microstructure, in terms of both the dentin–sealer interface and the inner mass. At the same time, the study revealed that, for BRS, the dentin–sealer area exhibited only small particle inclusions, with a different appearance to the large particles observed in the inner mass of the sealer (Figure 6D). This aspect was also described by Keboudi-Benezra [36]. Regarding the bioceramic sealers, SEM investigation showed a band of structurally altered dentin, a result of the sealer penetrating the dentinal tubules in proximity to the main canal. This “mineral infiltration zone” was noticeable on the surfaces of all examined specimens filled with bioceramic sealers (Figure 6B1–D1) The existence of this layer has been described in previous studies [13,56], where the authors suggested that its presence is due to hydroxyapatite formation at the interfacial region of the two substrates.

Tubule penetration enhances the contact area between dentin and root filling materials, improving the sealing of the root canal system. Nonetheless, our study revealed, consistent with previous research performed by Schmidt et al. [50] (TotalFill BC sealer), Song et al. [57] (EndoSeal MTA), and Atmeh et al. [56] (BioRoot), that the depth of extension for bioceramic sealers is relatively modest, with no differences between tested materials. RSSE penetrated more deeply into the dentin tubules in comparison to the bioceramic sealers. All the same, the force required to dislocate the root canal obturation was not dependent on the depth of its penetration into the dentinal tubules, as also observed by Tedesco [58] and De-Deus [51] in their studies.

## 5. Conclusions

Within the limitations of this in vitro study, it can be concluded that bioceramic endodontic sealers had a higher bond strength to root dentin than methacrylate resin-based endodontic sealer. Among the three bioceramic sealers that were assessed, Endoseal MTA achieved the most effective adhesion.

The use of bioceramic sealers in association with a single gutta-percha cone adapted to canal preparation may represent an adequate option for endodontic filling. However, additional experimental research is necessary to observe the interaction between bioceramic sealers and radicular dentin under variable conditions (e.g., irrigation solutions, master cone dimensions, and age of patient). These studies may provide valuable information that could improve the clinical performance of these materials.

## Figures and Tables

**Table 1:** Inclusion and exclusion criteria for selected teeth.

Criteria	Teeth
Inclusion	Intact root surface
	Complete root formation
	No signs of internal or external resorptions
	Circular root canal
	Degree of curvature less than 10°
	The size of the root canal near the apical foramen allowed the insertion of a file ISO 15
Exclusion	Teeth with two root canals
	Teeth with cracks
	Teeth with resorptions or caries
	Previous root canal treatment

**Table 2:** Chemical composition of tested endodontic sealers.

Sample	Delivery Form	Sample Composition
RealSeal SE (SybronEndo, Orange, CA, USA) (RSSE)	Two-paste system	Bisphenol glycidyl dimethacrylate (BISGMA), urethane dimethacrylate (UDMA), polyethylene glycol dimethacrylate (PEGDMA), ethoxylated bisphenol-A dimethacrylate (EBPADMA), barium sulfate, barium borosilicate glass, silica, calcium hydroxide, bismuth oxychloride with amines, aluminum oxides
EndoSequence BC Sealer (Brasseler USA, Savannah, GA, USA) (EBCS)	Single-paste system	Tricalcium silicate, dicalcium silicate, calcium phosphate monobasic, calcium hydroxide, zirconium oxide, tantalum oxide
Endoseal MTA (Maruchi, Wonju, Korea) (EDS)	Single-paste system	Calcium silicates, calcium aluminates, calcium aluminoferrite, calcium sulfates, zirconium dioxide, bismuth trioxide
Bioroot RCS (Septodont, St.Maur-des-Fossés, France) (BRS)	Powder–liquid system	Tricalcium silicate, zirconium oxide, povidone, aqueous solution of calcium chloride and polycarboxylate

**Table 3:** Push-out strength values (MPa) of tested endodontic sealers.

Materials	Number of Samples	Mean ± SD	Min	Max	Median
RSSE	60	1.059 ± 1.240	0.150	4.581	0.569
EBCS	60	2.803 ± 1.756	0.306	7.791	2.645
EDS	60	4.092 ± 2.232	1.405	11.233	3.881
BRS	60	2.038 ± 1.672	0.222	5.873	1.752

**Table 4:** Mean and standard deviation of push-out strength values (MPa) within the apical, middle, and coronal thirds.

Materials	Apical (Sections 1, 2)	Middle (Sections 3, 4)	Coronal (Sections 5, 6)
RSSE	0.799 ± 0.807	1.568 ± 1.692	0.637 ± 0.876
EBCS	3.366 ± 1.650	3.301 ± 1.458	1.928 ± 1.798
EDS	4.467 ± 1.548	4.54 ± 2.916	3.584 ± 2.001
BRS	2.598 ± 2.156	2.218 ± 1.145	1.370 ± 1.236

**Table 5:** Distribution of failure mode during dislodgement for tested sealers (as a percentage).

Materials	Adhesive	Mixed	Cohesive
RSSE	64.28	21.42	14.28
EBCS	9.52	23.80	66.66
EDS	5.55	22.22	72.22
BRS	63.15	10.52	26.31

## Data Availability

The data presented in this study are available on request from the corresponding author.

## References

[1] Holland R., Gomez Filho J.E., Cintra L.T.A., De Azevedo Queiroz I.O., Estrela C. (2017). Factors affecting the periapical healing process of endodontically treated teeth. J. Appl. Oral Sci..

[2] Kombayashi T., Colmenar D., Cvach N., Bhat A., Primus C., Imai Y. (2020). Comprehensive review of current endodontic sealers. Dent. Mat. J..

[3] Eltair M., Pitchika V., Hickel R., Kühnisch J., Diegritz C. (2018). Evaluation of the interface between gutta-percha and two types of sealers using scanning electron microscopy (SEM). Clin. Oral Investig..

[4] Tay F.R., Pashley D.H. (2007). Monoblocks in root canals: A hypothetical or a tangible goal. J. Endod..

[5] Tay F.R., Hiraishi N., Pashley D.H., Loushine R.J., Weller N., Gillespie W.T., Doyle M.D. (2006). Bondability of Resilon to a methacrylate-based root canal sealer. J. Endod..

[6] Stoll R., Thull P., Hobeck C., Yüksel S., Jablonski-Momeni A., Roggendorf M.J., Frankenberger R. (2010). Adhesion of self-adhesive root canal sealers on gutta-percha and Resilon. J. Endod..

[7] Hiraishi N., Papacchini F., Loushine R.J., Weller R.N., Ferrari M., Pashley D.H., Tay F.R. (2005). Shear bond strength of Resilon to a methacrylate-based root canal sealer. Int. Endod. J..

[8] Sfeir G., Zogheib C., Patel S., Giraud T., Venkateshbabu N., Bukiet F. (2021). Calcium Silicate-Based Root Canal Sealers: A Narrative Review and Clinical Perspectives. Materials.

[9] Camilleri J., Montesin F.E., Brady K., Sweeney R., Curtis R.V., Ford T.R. (2005). The constitution of mineral trioxide aggregate. Dent. Mater..

[10] Watson T.F., Atmeh A.R., Sajini S., Cook R.J., Festy F. (2014). Present and future of glass-ionomers and calcium-silicates cements as bioactive materials in dentistry: Biophotonics-based interfacial analyses in health and disease. Dent. Mater..

[11] Seo D.-G., Lee D., Kim Y.-M., Song D., Kim S.-Y. (2019). Biocompatibility and Mineralization Activity of Three Calcium Silicate-Based Root Canal Sealers Compared to Conventional Resin-Based Sealer in Human Dental Pulp Stem Cells. Materials.

[12] Jafari F., Jafari S. (2017). Composition and physicochemical properties of calcium silicate based sealers: A review article. J. Clin. Exp. Dent..

[13] Reyes-Carmona J.F., Felippe M.S., Felippe W.T. (2009). Biomineralization ability and interaction of mineral trioxide aggregate and white Portland cement with dentin in a phosphate-containing fluid. J. Endod..

[14] Viapiana R., Moinzadeh A.T., Camilleri L., Wesselink P.R., Tanomaru F., Camilleri J. (2016). Porosity and sealing ability of root fillings with gutta-percha and BioRoot RCS or AH Plus sealers. Evaluation by three ex vivo methods. Int. Endod. J..

[15] Jung S., Libricht V., Sielker S., Hanisch M.R., Schafer E., Dammaschke T. (2019). Evaluation of the biocompatibility of root canal sealers on human periodontal ligament cells ex vivo. Odontology.

[16] Camps J., Jeanneau C., El Ayachi I., Laurent P., About I. (2015). Bioactivity of a Calcium Silicate-based Endodontic Cement (BioRoot RCS): Interactions with Human Periodontal Ligament Cells In Vitro. J. Endod..

[17] Roizenblit R.N., Soarez F.O., Lopez R.T., Dos Santos B.C., Gusman H. (2020). Root canal filling quality of mandibular molars with EndoSequence BC and AH Plus sealers: A micro-CT study. Aust. Endod. J..

[18] Donnermeyer D., Ibing M., Bürklein S., Weber I., Reitze M.P., Schäfer E. (2021). Physico-Chemical Investigation of Endodontic Sealers Exposed to Simulated Intracanal Heat Application: Hydraulic Calcium Silicate-Based Sealers. Materials.

[19] Silva E.J., Carvalho N.K., Prado M.C., Zanon M., Senna P.M., Souza E.M., De-Deus G. (2016). Push-out Bond Strength of Injectable Pozzolan-based Root Canal Sealer. J. Endod..

[20] Yoo Y.-J., Baek S.-H., Kum K.-Y., Shon W.-J., Woo K.-M., Lee W. (2016). Dynamic intratubular biomineralization following root canal obturation with pozzolan-based mineral trioxide aggregate sealer cement. Scanning.

[21] Lin G.S.S., Ghani N.R.N.A., Noorani T.Y., Ismail N.H., Mamat N. (2021). Dislodgement resistance and adhesive pattern of different endodontic sealers to dentine wall after artificial ageing: An in-vitro study. Odontology.

[22] Chen H., Zhao X., Qiu Y., Xu D., Cui L., Wu B. (2017). The tubular penetration depth and adaptation of four sealers: A scanning electron microscopic study. Biomed. Res. Int..

[23] Ballullaya S.V., Vinay V., Thumu J., Devalla S., Bollu I.P., Balla S. (2017). Stereomicroscopic dye leakage measurement of six different root canal sealer. J. Clin. Diagn. Res..

[24] Mandhuri G.V., Varri S., Bolla N., Mandava P., Akkala L.S., Shaik J. (2016). Comparison of bond strength of different endodontic sealer to root dentin:an in vitro push-out test. J. Consev. Dent..

[25] Neelakantan P., Subbarao C., Subbarao C.V., De-Deus G., Zehnder M. (2011). The impact of root dentine conditioning on sealing ability and push-out bond strength of an epoxy resin root canal sealer. Int. Endod. J..

[26] Sagsen B., Ustun Y., Demirbuga S., Pala K. (2011). Push-out bond strength of two new calcium silicates-based endodontic sealers to root canal dentine. Int. Endod. J..

[27] Brichko J., Burrow M., Parashos P. (2018). Design Variability of the Push-out Bond Test in Endodontic Research: A Systematic Review. J. Endod..

[28] Schneider S.W. (1971). A comparison of canal preparation in straight and curved root canal. Oral Surg. Oral Med. Oral Pathol. Oral Radiol..

[29] Stelzer R., Schaller H.G., Gernhardt R. (2014). Push-out Bond Strength of RealSeal SE and AH Plus after Using Different Irrigation Solutions. J. Endod..

[30] Pane E.S., Palamara J.E.A., Messer H.H. (2013). Critical evaluation of push-out test for root canal filling materials. J. Endod..

[31] Collares F.M., Portella F.F., Rodrigues S.B., Celeste R.K., Leitune V.C.B., Samuel S.M.W. (2015). The influence of methodological variables on the push-out resistance to dislodgement of root filling materials: A meta-regression analysis. Int. Endod..

[32] Nagas E., Uyanik O., Durmaz V., Cehreli Z.C. (2011). Effect of pluger diameter on the push-out bond values of different root filing materials. Int. Endod. J..

[33] Chen W.-P., Chen Y.-Y., Huang S.-H., Lin C.-P. (2013). Limitations of push-out test in bond strength measurement. Endod. J..

[34] Donnemeyer D., Dornseifer P., Schaffer E., Dammaschke T. (2018). The push-out bond strength of calcium silicate-based endodontic sealer. Head Face Med..

[35] Retana-Lobo C., Tanomaru-Filho M., Guerreiro-Tanomaru J.M., Benavides-García M., Hernández-Meza E., Reyes-Carmona J. (2021). Push-Out Bond Strength, Characterization, and Ion Release of Premixed and Powder-Liquid Bioceramic Sealers with or without Gutta-Percha. Scanning.

[36] Kebudi Benezra M., Schembri Wismayer P., Camilleri J. (2018). Interfacial characteristics and cytocompatibility of hydraulic sealer cements. J. Endod..

[37] De-Deus G., Oliveira D.S., Cavalcante D.M., Simões-Carvalho M., Belladonna F.G., Antunes L.S., Souza E.M., Silva E.J.N.L., Versiani M.A. (2021). Methodological proposal for evaluation of adhesion of root canal sealers to gutta-percha. Int. Endod..

[38] Pawar A.M., Pawar S., Kfir A., Pawar M., Kokate S. (2016). Push-out bond strength of root fillings made with C-Point and BC sealer versus gutta-percha and AH Plus after the instrumentation of oval canals with the Self-Adjusting File versus WaveOne. Int. Endod. J..

[39] Gade V.J., Belsare L.D., Patil S., Bhede R., Gade J.R. (2015). Evaluation of push-out bond strength of endosequence BC sealer with lateral condensation and thermoplasticized technique: An in vitro study. J. Cons. Dent..

[40] Falakaloğlu S., Gündoğar M. (2022). Evaluation of the push out of bond strength of different bioceramic root canal sealers with different obturation techniques. Giornale di Endodontia.

[41] Miletic I., Chieffi N., Rengo C., Ferrari M., Nathanson D., Baraba A. (2016). Effect of photon induced photoacoustic streaming (PIPS) on bond strength to dentine of two root canal filling materials. Laser Surg. Med..

[42] Stiegemeier D., Baumgarther J.C., Ferracane J. (2010). Comparison of push-out bond strengths of Resilon with three different sealers. J. Endod..

[43] Kim Y.K., Mai S., Haycock J.R., Kim S.K., Loushine R.J., Pashley D.H., Tay F.R. (2009). The self-etching potential of RealSeal versus RealSeal SE. J. Endod..

[44] Ehsani S., Bolhari B., Etemadi A., Ghorbanzadeh A., Sabet Y., Nosrat A. (2013). The effect of Er,Cr:YSGG laser irradiation on the push-out bond strength of RealSeal Self-Etch sealer. Photomed. Laser Surg..

[45] Dickens S.H., Cho B.H. (2005). Interpretation of bond fallure through conversion and residual solvent measurements and Weibull analyses of flexural and microtensile bond strengths of bonding agents. Dent. Mater..

[46] Hammad M., Qualtrough A., Silikas N. (2008). Extended setting shrinkage behavior of endodontic sealers. J. Endod..

[47] Fuzinatto R.N., Farina A.P., Souza M.A., Miyagaki D.C., Randi Ferraz C.C., Cecchin D. (2017). Effects of an endodontic auxiliary chemical substance on the bond strength of two methacrylate-based endodontic sealers to dentin. Res. Tech..

[48] Braga R.R., Ferracane J.L. (2002). Contraction stress related to degree of conversion and reaction kinetics. J. Dent. Res..

[49] Mahdi A.A., Bolaños-Carmona V., Gonzalez-Lopez S. (2013). Bond strength to root dentin and fluid filtration test of AH Plus/gutta-percha, EndoREZ and RealSeal systems. J. Appl. Oral Sci..

[50] Schmidt S., Schafer E., Burklein S., Rohrbach A., Donnermeyer D. (2021). Minimal dentinal tubule penetration of endodontic sealers in warm vertical compaction by direct detection via SEM analysis. J. Clin. Med..

[51] De-Deus G., Brandão M.C., Souza E.M., Reis C., Reis K., Machado R., Neelakantan P. (2017). Epoxy Resin-Based Root Canal Sealer Penetration into Dentin Tubules Does not Improve Root Filling Dislodgement Resistance. Eur. Endod. J..

[52] Manolea H., Antoniac I., Miculescu M., Rica R., Platon A., Melnicenco R. (2016). Variability of the composite resins adhesion with the dental substrate preparation and the used adhesive type. J. Adhes. Sci. Technol..

[53] Antoniac A., Sinescu C., Antoniac A. (2016). Adhesion aspects in biomaterials and medical devices. J. Adhes. Sci. Technol..

[54] Bors A., Antoniac I., Cotrut C., Antoniac A., Szekely M. (2016). Surface analysis of contemporary aesthetic dental filling materials after storage in erosive solutions. Mater. Plast..

[55] Earar K., Antoniac V.I., Baciu S., Bran S., Onisor F., Milea C., Mohan A., Grigoroiu R., Saceleanu A., Manole M. (2017). Etching treatment effect on surface morphology of dental structures. Rev. Chim..

[56] Atmeh A.R., Chong E.Z., Richard G., Festy F. (2012). Watson T F. Dentin-cement Interfacial Interaction: Calcium Silicates and Polyalkenoates. J. Dent. Res..

[57] Song D., Yang S.-E. (2022). Comparison of dentinal tubule penetration between a calcium silicate-based sealer with ultrasonic activation and an epoxy resin-based sealer: A study using confocal laser scanning microscopy. Eur. J. Dent..

[58] Tedesco M., Chain M.C., Felippe W.T., Alves A.M.H., Garcia L.D.F.R., Bortoluzzi E.A., Cordeiro M.R., Teixeira C.S. (2019). Correlation between Bond Strength to Dentin and Sealers Penetration by Push-Out Test and CLSM Analysis. Braz. Dent. J..

